# Supplementary material for: Genome-wide association studies reveal stable loci for wheat grain size under different sowing dates
Source: PeerJ. 2024 Feb 26;12:e16984. doi: 10.7717/peerj.16984 (PMC10903348; doi:10.7717/peerj.16984)
Supplement: Figure S1 — Blue font represents accessions and brown font represents environments. [file peerj-12-16984-s001.pdf]

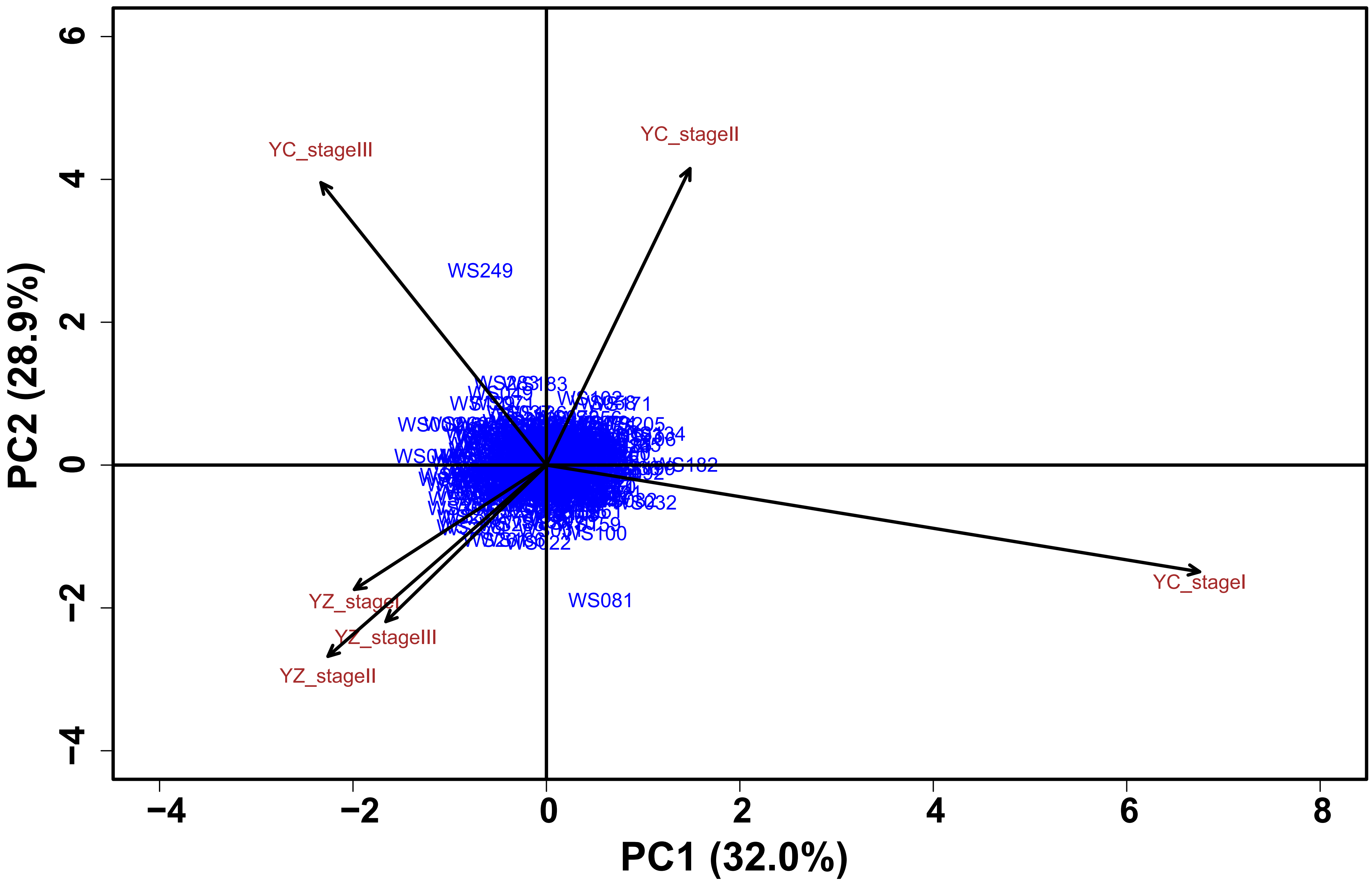

YC_stageIII
YC_stageII
YC_stageI
YZ_stageI
YZ_stageIII
YZ_stageII
WS249
WS081
WS182
WS232
WS100
PC1 (32.0%)
PC2 (28.9%)
−4
−2
0
2
4
6
8
