# Supplementary figures and images for: Genome-wide association studies reveal stable loci for wheat grain size under different sowing dates
Source: PeerJ. 2024 Feb 26;12:e16984. doi: 10.7717/peerj.16984 (PMC10903348; doi:10.7717/peerj.16984)

**A**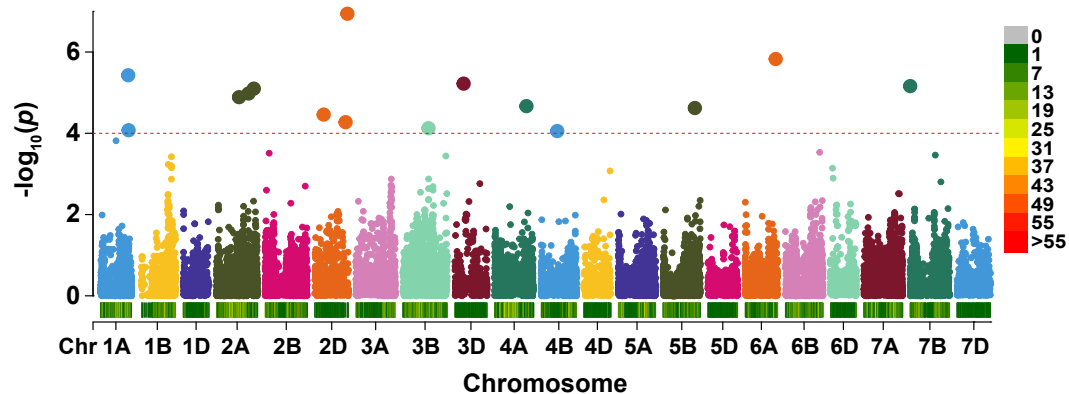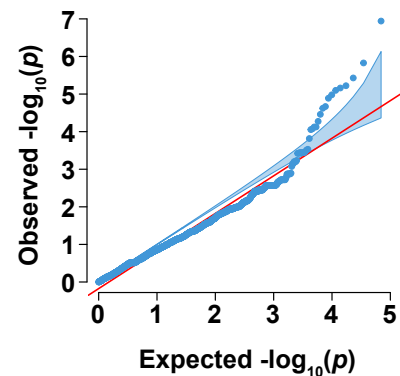**B**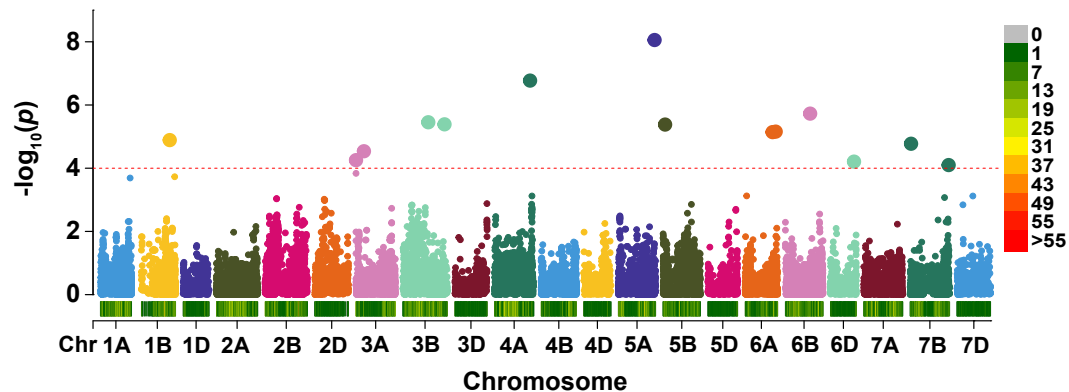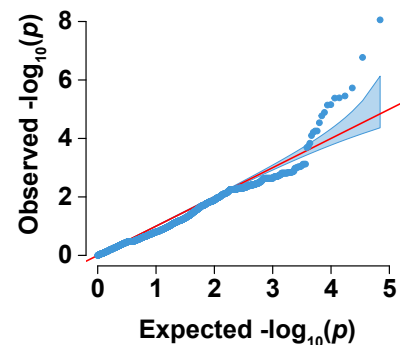**C**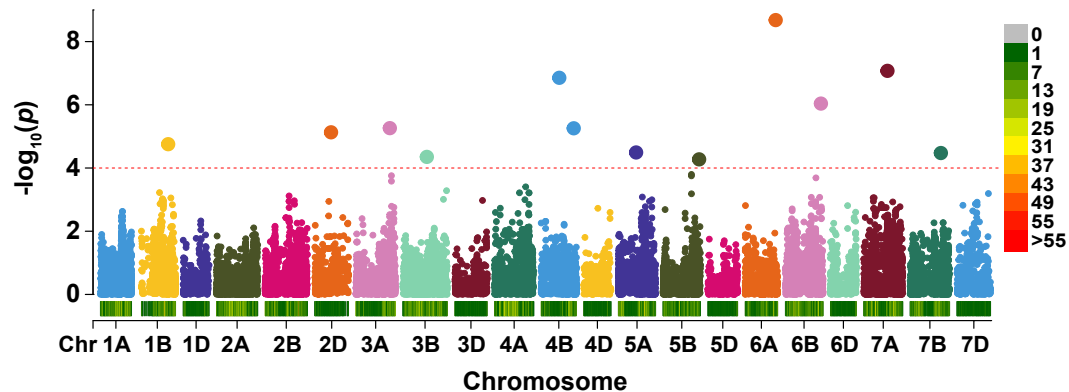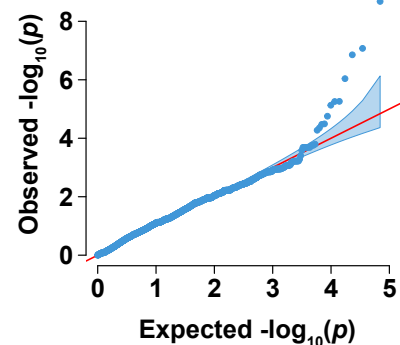

Supplement: Figure S2 — Manhattan and QQ plots for grain length (GL), grain width (GW), and thousand-grain weight (TGW) are shown in (A-C), respectively. The horizontal red lines represent the threshold. [file peerj-12-16984-s002.pdf]

**A**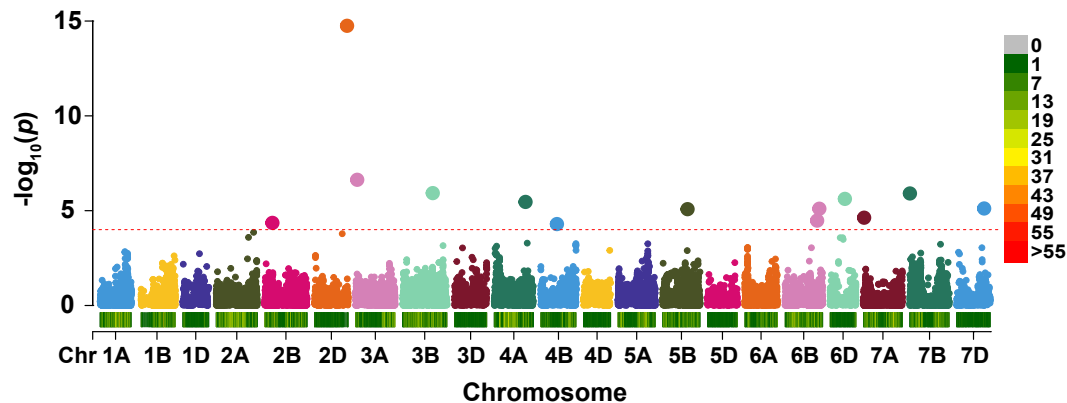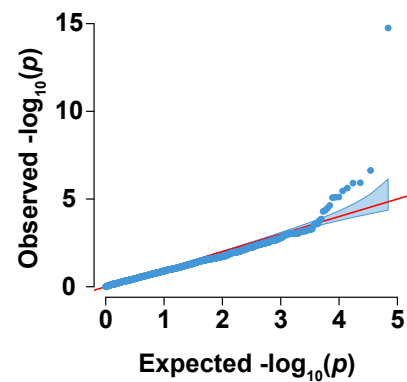**B**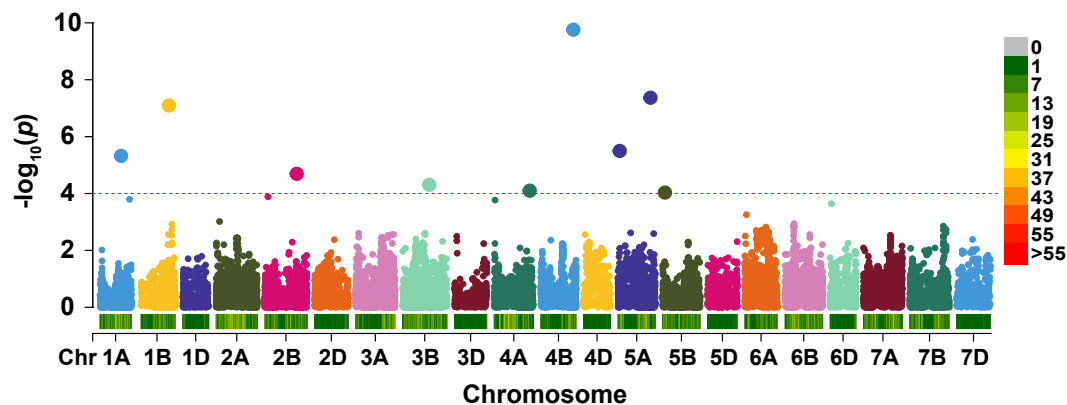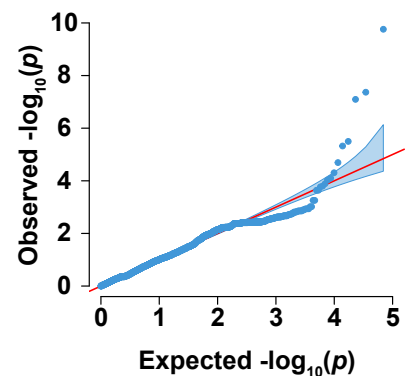**C**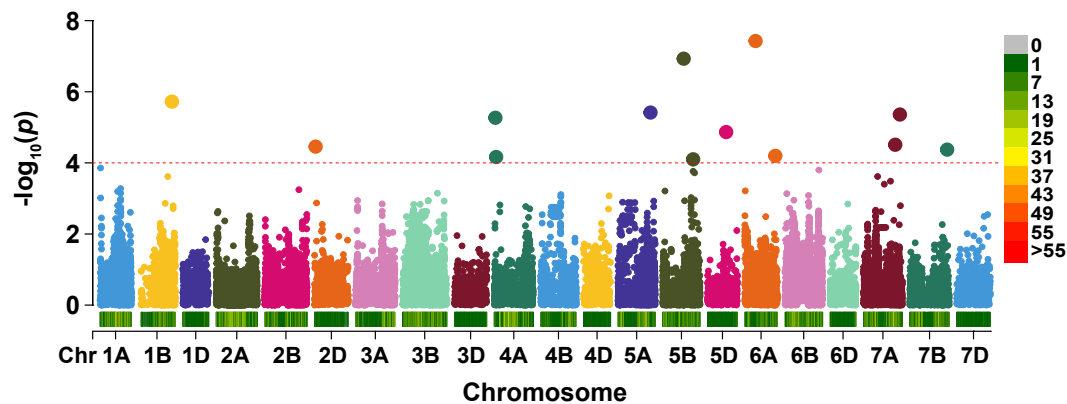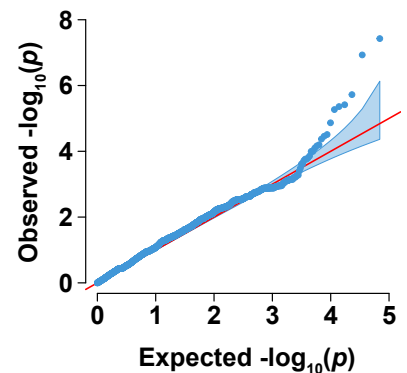

Supplement: Figure S3 — Manhattan and QQ plots for grain length (GL), grain width (GW), and thousand-grain weight (TGW) are shown in (A-C), respectively. The horizontal red lines represent the threshold. [file peerj-12-16984-s003.pdf]

**A**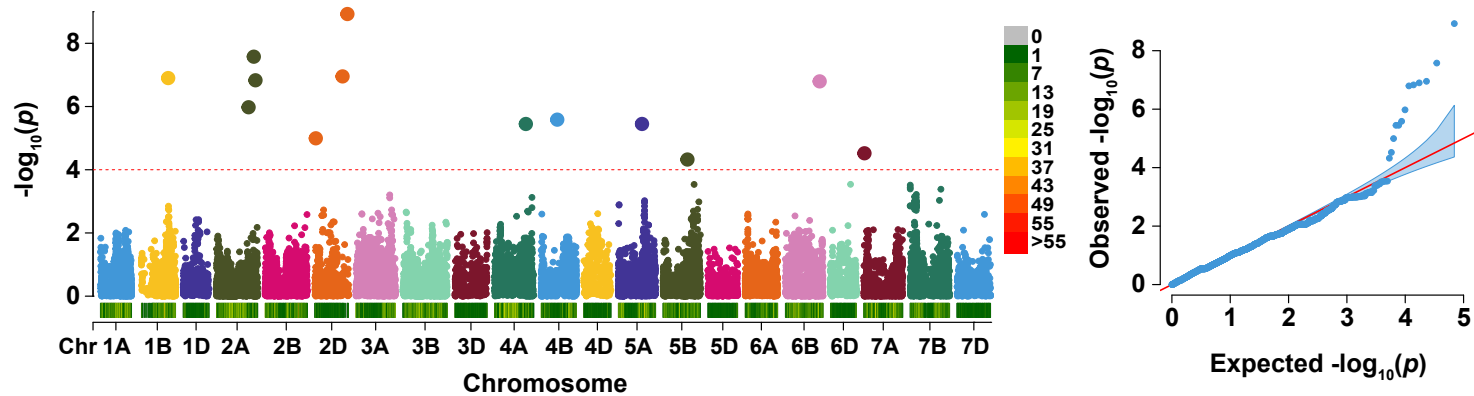**B**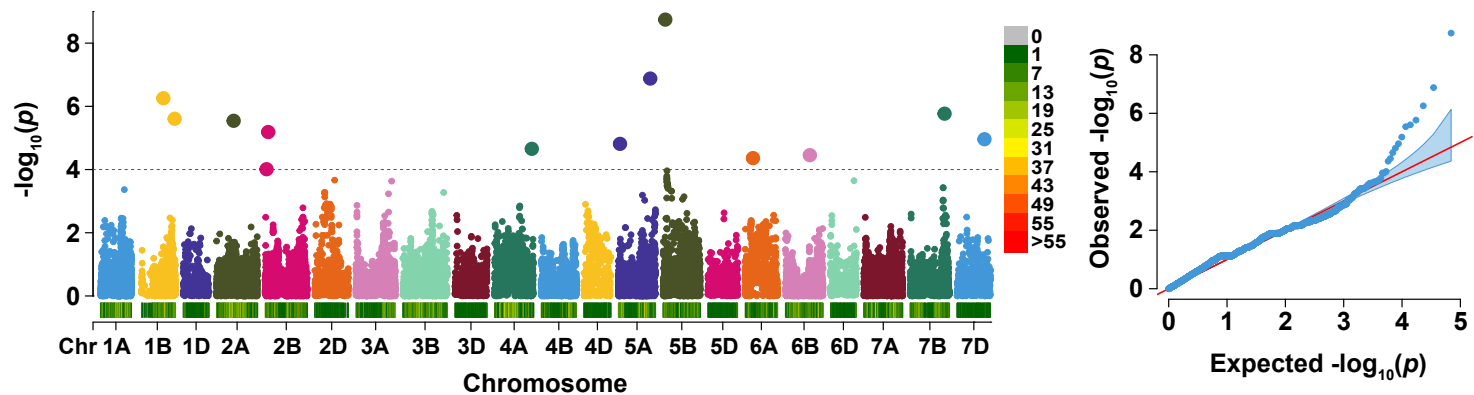**C**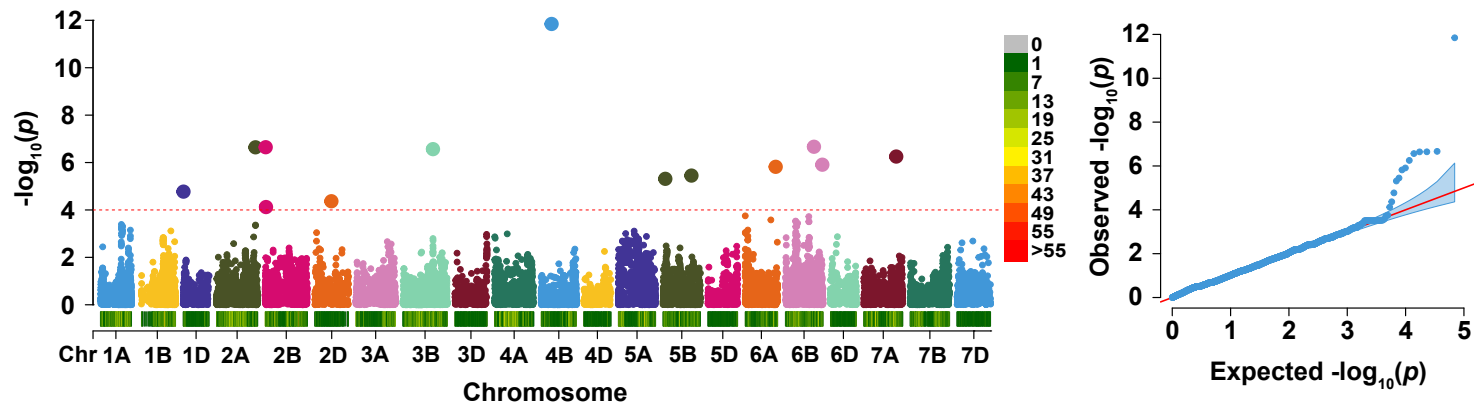

Supplement: Figure S4 — Manhattan and QQ plots for grain length (GL), grain width (GW), and thousand-grain weight (TGW) are shown in (A-C), respectively. The horizontal red lines represent the threshold. [file peerj-12-16984-s004.pdf]
